# Identification of Novel Interacting Proteins of FUZ and GPR161

**DOI:** 10.1002/pmic.70164

**Published:** 2026-07-04

**Authors:** Gabriella Salazar, Anna Battenhouse, Andrew J. Kim, Sung‐Eun Kim

**Affiliations:** ^1^ Department of Pediatrics Dell Pediatric Research Institute Dell Medical School The University of Texas at Austin Austin Texas USA; ^2^ Biomedical Research Computing Facility The University of Texas at Austin Austin Texas USA

**Keywords:** affinity‐based mass spectrometry, FUZ, GPR161, protein interactome

## Abstract

Protein–protein interactions are central to the dynamic regulation of signaling pathways and provide critical insight into the cellular mechanisms underlying human disease. Our previous study demonstrated biochemical and genetic interactions between FUZ and GPR161 in sonic hedgehog signaling during spinal neural tube development. In this study, we sought to identify novel interacting proteins of FUZ and GPR161 to further characterize their biochemical and functional relationships. Using affinity‐based liquid chromatography–tandem mass spectrometry of immunoprecipitated complexes from cells overexpressing FUZ and GPR161, we identified 159 shared co‐interacting proteins, along with 289 proteins exclusive to FUZ and 617 proteins exclusive to GPR161. Gene Ontology (GO) analysis of the co‐interactome revealed significant enrichment in proteasomal catabolic processes and intracellular trafficking pathways. GO analysis of the FUZ‐specific interactome showed enrichment in cell cycle progression, mitochondrial membrane, and RNA metabolism, whereas the GPR161‐specific interactome was enriched in receptor complex and endoplasmic reticulum‐Golgi transport pathways. These findings were supported by STRING network analysis. Among the prioritized candidates, FKBP8 was validated as a binding partner of both FUZ and GPR161. Collectively, our proteomic study defines the protein interaction networks of FUZ and GPR161, highlighting their distinct and cooperative roles in multiple cellular processes and providing a foundation for future functional studies.

## 1 Introduction

Protein–protein interaction (PPI) involves physical and biochemical interactions between proteins in a functional domain‐dependent manner. Protein complexes regulated by PPI control multiple cellular processes in a context‐dependent manner. Most PPIs are transient and specific in response to extracellular stimuli and the conditions of adjacent proteins. This nature of PPI allows multiple signaling pathways to transduce specific commands to cells for appropriate cellular outcomes. In addition, genetic mutations linked to human diseases inevitably cause protein dysfunction, regardless of whether they are coding or noncoding mutations. One of the landmarks of functional defects in proteins due to genetic mutations is disrupted PPI. For instance, compromised PPI due to genetic modification of candidate genes in neural tube defects (NTDs) has been widely investigated [1, 2, 3] to understand the functional role of individual proteins resulting from mutated genes found in NTD patients. Collectively, a comprehensive PPI network allows us to better understand human diseases.

**Significance Statement**

The molecular relationship between FUZ and GPR161 remains poorly defined; here we sought to characterize their shared and distinct protein interactome to define their functional connection at the protein level.

Using affinity‐based mass spectrometry integrated with gene ontology and STRING network analysis, we systematically mapped the protein interactomes of FUZ and GPR161, revealing novel molecular association and candidate interaction partners.

We prioritized FKBP8 as a co‐interacting partner of both FUZ and GPR161 and validated their association biochemically. Together, FUZ, GPR161, and FKBP8 form a trimeric protein complex, and their interactions were nonexclusive, suggesting that they co‐exist within a shared protein network, such as signaling pathway.

G‐protein‐coupled receptor 161 (GPR161) is a G protein‐coupled receptor (GPCR), which is the largest receptor family in the human proteome. GPR161 localizes to primary cilia to function as a negative regulator of the sonic hedgehog (Shh) signaling pathway via the Gas‐cAMP‐PKA axis [4]. As the ciliary localization of GPR161 is critical for its basal suppressive activity on Shh signaling via regulating Gli2 and Gli3 processing, trafficking in and out of the primary cilia is the key process to determine GPR161 activity. In addition, GPR161 acts as an A‐kinase anchored protein (AKAP), thereby facilitating the compartmentalization of GPR161 into the plasma membrane and spatiotemporal PKA recruitment to GPR161 [5]. Therefore, dynamic PPI is required for proper GPR161 activity to regulate Shh signaling pathway. Loss‐of‐function and dominant negative mutations of *GPR161* in humans and mice are associated with several pediatric diseases, including multiple birth defects, pediatric tumors, and pituitary stalk interruption syndrome [6, 7, 8, 9, 10, 11, 12]. However, its associated protein network and its other effector pathways other than Shh signaling have not been fully investigated.

The Fuzzy planar cell polarity protein, FUZ, is a Ciliogenesis and PLANar polarity effector (CPLANE) protein complex that is critical for proper ciliogenesis [13, 14]. FUZ also facilitates retrograde ciliary trafficking as a retrograde IFT complex [15]. Loss‐of‐function mutants of *Fuz* in mice present with NTDs, limb abnormalities, and craniofacial defects [13, 16]. Mutations in the FUZ gene in humans are linked to NTDs, oral‐facial‐digital syndrome, and craniosynostosis [17, 18, 19]. Considering the role of primary cilia in Shh signaling and the range of phenotypic malformations in *Fuz* mutants in humans and mice, Fuz is also considered a part of Shh signaling [20], although its role has not been well characterized. Our recent work [21] revealed a genetic and biochemical link between FUZ and GPR161 in the regulation of Shh signaling in the context of spinal neural tube development. While we showed the evidence that FUZ may be involved in GPR161 trafficking within primary cilia [21], their biochemical and functional links have not been fully elucidated.

In this study, we identified the proteins that interact with FUZ and GPR161, as well as the proteins that interact with both FUZ and GPR161, using affinity purification‐mass spectrometry. Furthermore, we validated the biochemical interactions among FUZ, GPR161, and the prioritized protein. Our results reveal novel protein networks involving both FUZ and GPR161 and provide unexplored functional roles for these two proteins.

## 2 Materials and Methods

### 2.1 DNA Plasmids

The lentiviral vector (*pLV‐eGFP*) and packaging vectors (*pRSV‐Rev, pMDLg/pRRE, and pMD2.G*) were obtained from Addgene. hGPR161 genes were in‐frame inserted into *pLV‐eGFP* with BamHI, and *hFUZ* genes were in‐frame inserted into *pLV‐eGFP* with XbaI and BamHI. The expression vectors for *hGPR161‐HA* [6], *hGPR161‐GFP, hFUZ‐mCherry* [21], *and hFKBP8‐GFP* [1] were described in previous publications.

### 2.2 Cell Culture and Transfection

HEK293 cells (ATCC‐CRL‐1573) and HEK293T cells (ATCC‐CRL‐3216) cells were cultured in Dulbecco's Modified Eagle Medium (DMEM) with 1% penicillin‐streptomycin (PS) and 10% Fetal Bovine Serum (FBS) at 37°C and 5% CO_2_. For overexpression studies, HEK293 and HEK293T cells were transfected with the respective DNA plasmids and Lipofectamine 2000 (Life Technologies) and incubated for 48 h, according to the manufacturer's instructions.

### 2.3 Lentivirus Production and Infection

For lentivirus production, 293T cells were transfected with *pLV‐eGFP*, *pLV‐GPR161‐eGFP*, or *pLV‐FUZ‐eGFP* together with *pRSV‐Rev*, *pMDLg/pRRE*, and *pMD2.G* using Lipofectamine 2000. The virus‐containing medium was collected 48 h post‐transfection, and fresh medium was added. The virus‐containing medium was collected every 12 h for two to three additional times. All collected virus‐containing media were centrifuged at 3000 rpm for 20 min at 4°C. The supernatant was collected and stored at −80°C until infection. For lentivirus infection, 293 cells were plated and infected with virus‐containing medium with 8 mg/ml polybrene for 24 h, followed by replacement with fresh media and incubation for an additional 24 h until lysis.

### 2.4 Co‐Immunoprecipitation

HEK293T and HEK293 cells were either transfected with expression vectors indicated or infected with lentivirus containing GFP, GPR161‐GFP, or FUZ‐GFP and lysed with digitonin‐based GPCR immunoprecipitation (GPCR IP) lysis buffer, as previously described [22]. Lysates were immunoprecipitated with ChromoTek GFP‐Trap agarose (Proteintech) at 4°C overnight, followed by washing with GPCR IP lysis buffer. For HA IP, lysates were immunoprecipitated with either GFP or HA antibody with protein G agarose at 4°C overnight, followed by washing with GPCR IP lysis buffer. For mass spectrometry, the agarose after IP was washed vigorously (extra washes for extended time) to reduce the nonspecific binding. The IP lysates were subjected to SDS‐PAGE, followed by western blotting (WB) or in‐gel digestion for mass spectrometry.

### 2.5 Western Blot

Whole cell lysates or immunoprecipitated lysates were subjected to SDS‐PAGE, followed by WB. The primary antibodies used were as follows: GFP (Cell signaling #2956; 1:1000), HA (Cell signaling #3724; 1:1000), and RFP (MBL PM005; 1:3000). The secondary antibodies used were IRDye 800CW goat antirabbit IgG and IRDye 680RD goat anti‐mouse IgG (Li‐COR). Images were obtained using an Odyssey scanner (Li‐COR).

### 2.6 Liquid Chromatography Mass Spectrometry (LC‐MS/MS)

Immunoprecipitated lysates were subjected to run on the pre‐cast 4%–20% gradient proteins gels (Bio‐Rad) for 10 min. Gels were stained with Coomassie staining solution (Bio‐Rad), were excised, and were diced into 1 mm gel cubes with ethanol‐washed blades for in‐gel trypsin digestion, as described by Goodman et al. [23]. Proteins were digested with trypsin, digested peptides were desalted C18 resin‐filled pipette tips (Thermo Scientific) following the manufacturer's instruction, and identified by LC‐MS/MS on a Thermo Ultimate 3000 RSLCnano UPLC and Orbitrap Fusion Tribrid mass spectrometer with a 2 h run time, followed by Sequest HT database search in Proteome Discoverer 2.2 and Scaffold validation in the UT Austin Biological Mass Spectrometry Facility using previously published method [24, 25].

### 2.7 Database Search

LC‐MS/MS raw files were obtained for protein A agarose (two biological replicates) GFP (three biological replicates), FUZ‐GFP (three biological replicates), GPR161‐GFP (three biological replicates) IP samples and were converted to mzXML format with MSconvert ([26], ProteoWizard, 2011) using standard parameters, then analyzed using X!Tandem ([27], 2010.10.01.1 version), MS‐GF+ ([28], v9517 (4/10/2013)) and Comet ([29], 2013.02 rev.0) to match mass spectra to peptide sequences. All data were searched against the UniProt human proteome, (UP000005640, UniProt release 2018_02, downloaded in February 2018). All searches employed a 10‐ppm mass tolerance for both precursor and fragment ions for higher energy collisional dissociation (HCD). Enzyme specificity was set fully specific to trypsin (cleavage sites were RK, cleavage side was C‐terminal) with up to two missed cleavages allowed. Oxidation of methionine was used as a common modification. Peptide matches from the three search engines were probabilistically integrated using MSblender ([30], downloaded from Github “MSblender_consistent” branch in February 2020) and assigned to proteins as spectral counts. Data were filtered to a 1% false discovery rate (FDR) at the protein level using two types of decoy searching with common contaminant sequences (e.g. keratin, bovine serum albumin) added and all protein sequences including contaminants reversed.

### 2.8 Bait/Prey Interaction Analysis

The SAINTexpress (v3.6.3) [31] software package was used to discriminate between bona fide protein–protein interactions in affinity purified mass spectrometry experiments from nonspecific background interactions as modeled by control purifications. SAINTexpress input files (bait, prey and interactions) were formatted according to recommendations in the original SAINT analysis protocol document [32]. Specifically: total spectral counts were used for quantification; spectral counts for the bait itself were removed from its own experiments; and the control experiments (GFP and protein A) were treated as separate baits rather than replicates of a control type. The saintexp‐spc runs were performed with the ‐R 2 command line option (use the 2 best‐scoring interactions when more than 2 replicate observations are available) [31]. The outputs from the SAINTexpress runs were combined and annotated. Significance levels of baits were determined with two parameters:at least 1 unique peptide spectral match (PSM) and SAINT Baysian false discovery rate (FDR) < = 0.05. Two significance subsets were further annotated: Significance 1 (Sig 1) refers to at least one unique PSM and FDR ≤ 0.01, and significance 2 (Sig 2) refers to at least one unique PSM and FDR ≤ 0.05. Co‐interacting proteins of FUZ and GPR161 were selected from baits with Sig 1 and Sig 2 from both FUZ and GPR161.

### 2.9 Functional Enrichment Analysis

Gene Ontology (GO) analysis was performed in R (v3.6.1) using the topGO package (v2.38.1). Sig 1 and Sig 2 interacting proteins were first converted to corresponding gene IDs using GENCODE v34 [33] human gene annotations. Three data subsets (FUZ‐only, GPR161‐only, and FUZ+GPR161) were analyzed separately for the 3 GO ontologies (Biological Process/BP, Molecular Function/MF and Cellular Component/CC) against the org.Hs.eg.db annotation database using topGO's classic algorithm and Fisher's Exact test in count data mode. Due to substantial overlap of genes among GO terms, GO enrichment tests are not statistically independent. Therefore, multiple hypothesis testing correction was not applied, and the reported p‐values are unadjusted *p*‐values. Instead, a stringent *p*‐value threshold (*p*<10^−3^) was used to identify enriched GO terms Consistent with the recommendations of the topGO R package, *p*‐values were calculated and evaluated independently for each GO term. Protein network analysis based on known functional enrichment was performed using web‐based STRING analysis (https://string‐db.org, version 12.0).

### 2.10 Protein Structure Modeling

The prediction of protein–protein interaction modeling was performed using Alpha fold 3 [34].

## 3 Results

Our previous study revealed that *Fuz* and *Gpr161* are biochemically interacting in HEK293 cells as well as genetically linked to the regulation of Shh signaling during mouse spinal neural tube development [21]. Since GPR161 is mainly localized in the primary cilia [4] and FUZ is known to localize in the basal body to regulate ciliogenesis [13], we speculate that their biochemical interaction may not be a direct protein–protein interaction. Hence, we sought to identify the protein interactomes of FUZ and GPR161 to understand their functional relationships and to explore underappreciated functions of each protein using proteomics approaches.

To this end, we performed affinity‐based liquid chromatography with tandem mass spectrometry (LC‐MS/MS) analysis using overexpressed FUZ and GPR161 proteins. The overall experimental scheme is shown in Figure 1, and the basic validation experiments, including validation of FUZ and GPR161 overexpression and IP efficiency, are shown in Figure S1. In brief, HEK293 cells were infected with lentivirus containing GFP, FUZ‐GFP, or GPR161‐GFP. Cell lysates were immunoprecipitated with GFP nanobody, which is the smallest antibody with high affinity and low nonspecific binding, to minimize non‐specific binding to the IgG antibody structure. In addition, we included two kinds of negative controls, (1) two protein A bead‐only control pulled down with cell lysates overexpressing either FUZ‐GFP or GPR161‐GFP, and three GFP‐Trap pulled down with cell lysates overexpressing GFP only. These vigorous negative controls enable to rule out the possibility of identifying nonspecific interacting proteins and background contaminants due to immunoprecipitation (IP), overexpression and protein tag artifacts. We used HEK293 cells, since they are human ciliated cells [35], and our initial biochemical interaction of GPR161 and FUZ was identified in these cells.

**FIGURE 1:**
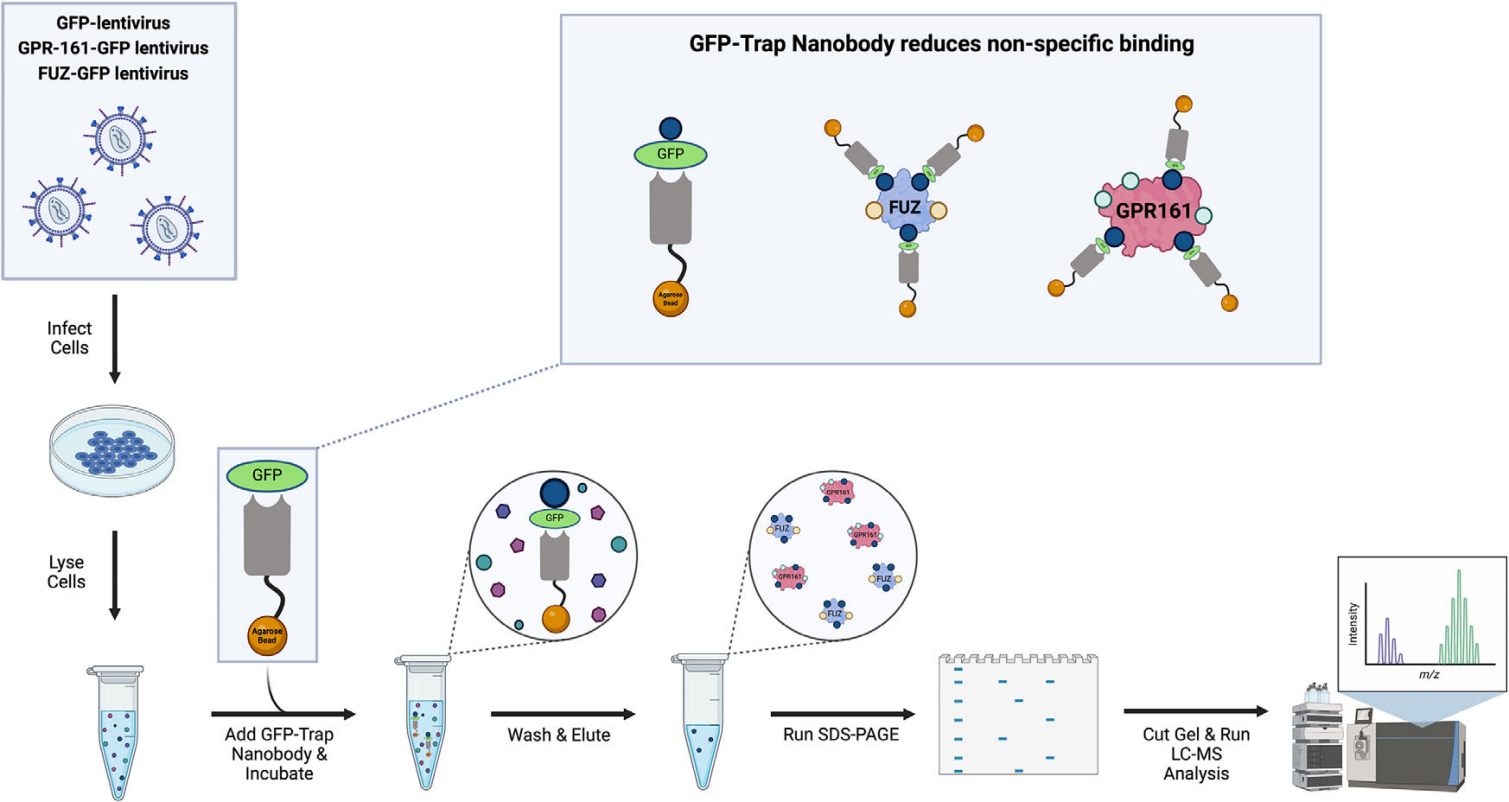
Overview of affinity purification‐based LC‐MS/MS experiments.293T cells were infected with lentivirus containing GFP, FUZ‐GFP, or GPR161‐GFP before lysis. The lysates were subjected to immunoprecipitation with GFP nanobody‐conjugated agarose to pull down the protein complex associated with GFP, FUZ‐GFP, or GPR161‐GFP proteins. After vigorous washing, the protein complexes with the antibody‐agarose complex were denatured, run on SDS‐PAGE, and cut for in‐gel digestion for mass spectrometry.

The LC‐MS/MS data were searched using the human UniProt database, and the peptide spectra counts were assigned to each protein. We identified proteins that significantly interacted with either FUZ or GPR161 using the Significance Analysis of INTeractome software (SAINTexpress analysis [31]). To identify the co‐interacting proteins for FUZ and GPR161, we sorted the proteins that included both FUZ and GPR161 interactomes with the highest significance based on SAINTexpress analysis. We identified a total of 776 proteins that significantly interacted with GPR161 and 448 proteins that interacted with FUZ. Among them, 159 proteins were identified to significantly interact with both GPR161 and FUZ (Figure 2A, Supporting Information). Of note, we identified a larger number of GPR161 interactome components than FUZ interactome components, even though the same controls and analytical criteria were used to remove background interactors.

**FIGURE 2:**
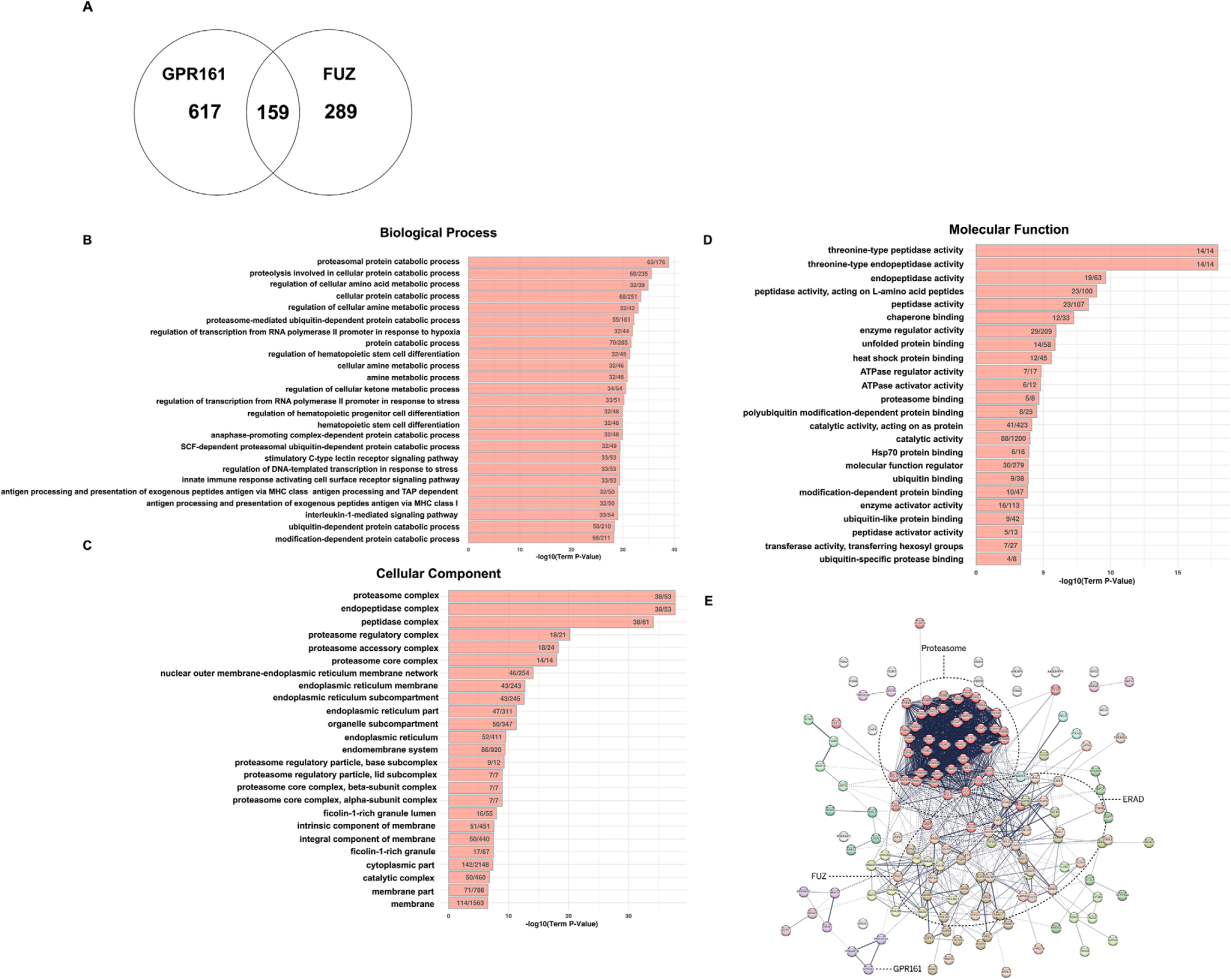
Identification and analysis of the co‐interactome of FUZ and GPR161(A) Venn diagram indicating the number of identified proteins that interact with both FUZ and GPR161 and exclusively interact with either FUZ or GPR161. (B‐D) Gene Ontology analysis of proteins that interact with both FUZ and GPR161. Top 25 protein categories for Biological Process (B), Cell Component (C), and Molecular Function ontologies (D). The numbers on each bar graph represent the number of proteins identified within each GO term category, relative to the total number of proteins in that category. Due to violation of the independent and identically distributed (IID) statistical requirement, FDR adjustment of p‐values was not performed; therefore, the reported p‐values are unadjusted, and a stringent p‐value threshold (*p* <10^−3^) was applied to each GO term. (E) STRING analysis of proteins interacting with both FUZ and GPR161. Proteins in the same category are color‐coded. Two main nodes, the proteasome and ERAD, are indicated by circles.

### 3.1 Analysis Co‐Interactome Between FUZ and GPR161

We first performed GO analysis to classify the FUZ and GPR161 co‐interactome to determine their functional enrichment (Figure 2B–D). The top five biological processes in the co‐interactome of FUZ and GPR161 included proteasomal protein catabolic pathways, proteolysis involved in the cellular protein catabolic process, regulation of the cellular amino acid metabolic process, cellular protein catabolic process, and regulation of the cellular amine metabolic process (Figure 2B). The top five cellular components were the proteasome complex, endopeptidase complex, peptidase complex, proteasome regulatory particle, and proteasome core complex (Figure 2C). The top five molecular functions were threonine‐type peptidase activity, threonine‐type endopeptidase activity, L‐amino acid peptidase activity, peptidase activity, and chaperone binding (Figure 2D). Intriguingly, the top terms in all three ontologies in the GO analysis of the FUZ–GPR161 co‐interactome were involved in functional processes relevant to protein degradation. To explore the functional association between individual proteins within the FUZ–GPR161 co‐interactome, we performed STRING analysis [36]. The proteins within the co‐interactome were functionally connected based on the existing protein interaction database in the STRING analysis (Figure 2E). Two large nodes within this connectome included the proteasome complex and ER‐associated degradation (ERAD) proteins (Figure 2E). There were 48 proteins in the proteasome complex and 31 proteins in the ERAD category, as shown in Figure S2. The notable overlap between the GO term analysis and our table reinforces the central role of the co‐interactome in protein processing and degradation. We then categorized 159 individual proteins into proteasomes, chaperones, ubiquitin‐dependent ligases and related proteins, trafficking proteins, enzymes relevant to post‐translational modification, and uncategorized proteins based on the protein functions described in the Uniprot protein database (Supplementary Table).

### 3.2 Analysis of FUZ Interactome

We examined proteins that exclusively interact with FUZ. As shown in Figure 1B, we identified 448 proteins that significantly interacted with FUZ. Among them, INTU, RSG, and OFD1 were included, which are known FUZ‐interacting proteins. We also compared the published FUZ interactome [14] to the FUZ interactome we identified, and there were 162 proteins present in both interactomes (Figure S3). We further examined 287 proteins that exclusively interacted with FUZ. Based on GO analysis, the top five biological processes included proteins enriched in mitotic cell cycle regulation and post‐transcriptional gene silencing (Figure 3A), which is consistent with their role in ciliogenesis. Proteins were enriched in the ubiquitin ligase complex and nucleoplasm in the top five cellular components and regulatory transcription factors, including repressors or binding, in the top five molecular functions (Figure 3A). We further examined the proteins that exclusively interact with FUZ using STRING analysis (Figure 3B). There were three large nodes within the FUZ interactome: RNA and nucleic acid metabolic processes (57 proteins), mitosis (55 proteins), and mitochondrial membrane organization (31 proteins) (Figure S4). Notably, mitotic proteins were annotated using both GO and STRING analyses.

**FIGURE 3:**
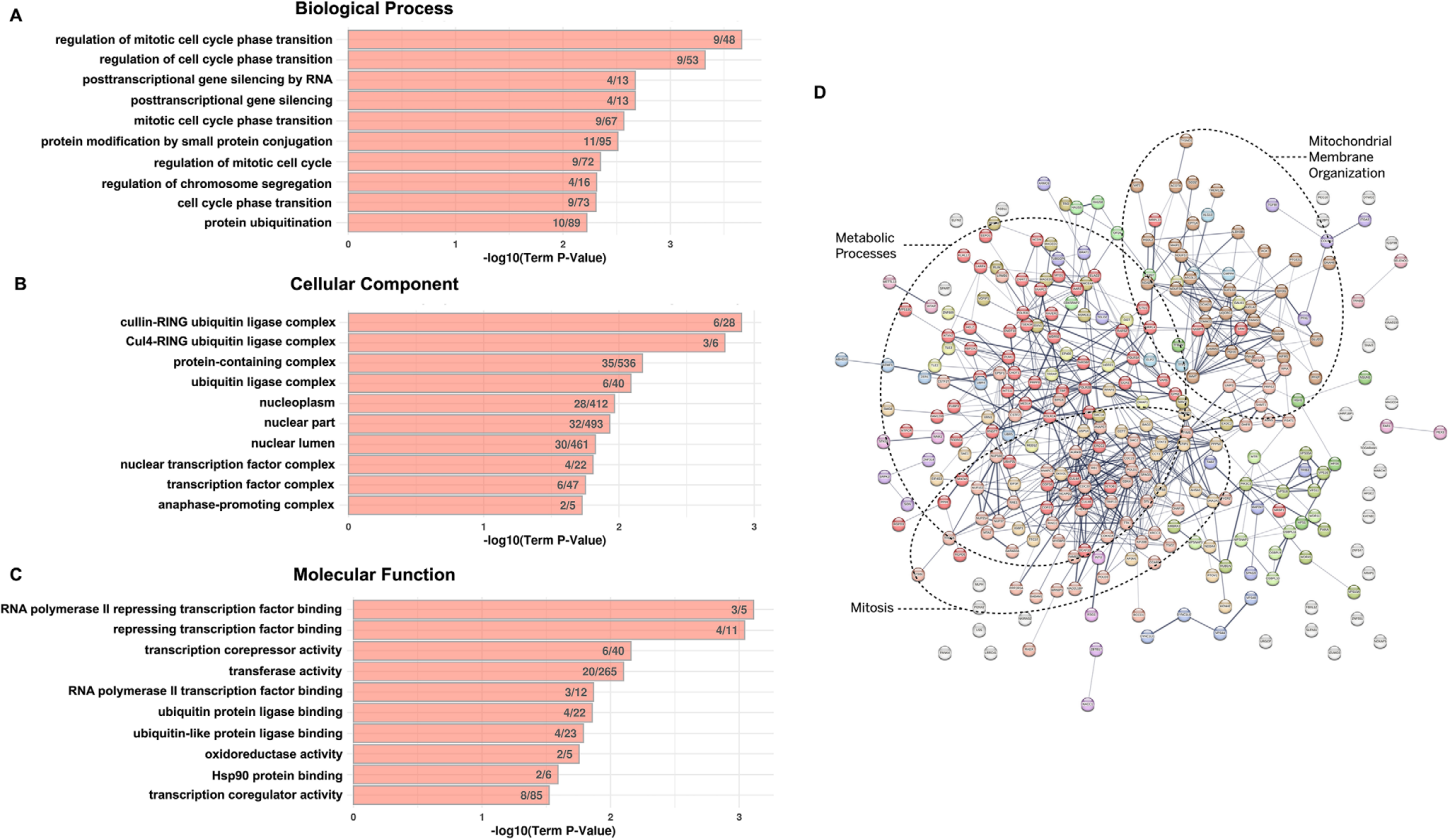
Identification and analysis of the exclusive interactome of FUZ.(A‐C) Gene Ontology analysis of FUZ‐exclusive interacting proteins. Top 10 protein categories for Biological Process (A), Cell Component (B), and Molecular Function ontologies (C). The numbers on each bar graph represent the number of proteins identified within each GO term category, relative to the total number of proteins in that category. Due to violation of the IID statistical requirement, FDR adjustment of p‐values was not performed; therefore, the reported p‐values are unadjusted, and a stringent p‐value threshold (*p* <10^−3^) was applied to each GO term. (D) STRING analysis of FUZ‐exclusive interacting proteins. Proteins in the same category are color‐coded. Three main nodes, metabolic process, mitosis, and mitochondrial membrane organization are indicated as circles.

### 3.3 Analysis of GPR161 Interactome

We analyzed the GPR161 interactome and found that PRKAR1A, PRKAR1B, AKAP4, and multiple PKA catalytic subunits (PRKACA, PRKACB, PRKACG, and PRKACSH) have been previously identified as GPR161 interacting proteins in the literatures [5, 37]. Among the GPR161 interactome, we identified 617 proteins that exclusively interacted with GPR161 (Figure 2A). The numbers of the GPR161 interactome were larger than those of the FUZ interactome, most likely due to its nature of GPCR, a receptor that can interact with various downstream effectors. Based on GO analysis, proteins were enriched in localization, glycoprotein metabolic processes, and transport in the Biological Process (Figure 4A). Proteins were enriched in membrane components in the top five cellular components (Figure 4B) and in transmembrane receptor protein kinase, molecular transducer, signaling receptor, and UDP‐glycosyltransferase activity in the top five molecular functions (Figure 4C). The GO results support the well‐known role of GPR161 as a GPCR. We then performed STRING analysis using the proteins that interacted exclusively with GPR161 (Figure 4D). Two main nodes were identified in the GPR161 interactome, including the enzyme‐linked receptor protein signaling pathway (136 proteins) and ER‐to‐Golgi vesicle‐mediated transport (71 proteins) (Figure S5). Enzyme‐linked receptor protein signaling pathways were identified by both STRING and GO analyses, suggesting the specific receptor function of GPR161.

**FIGURE 4:**
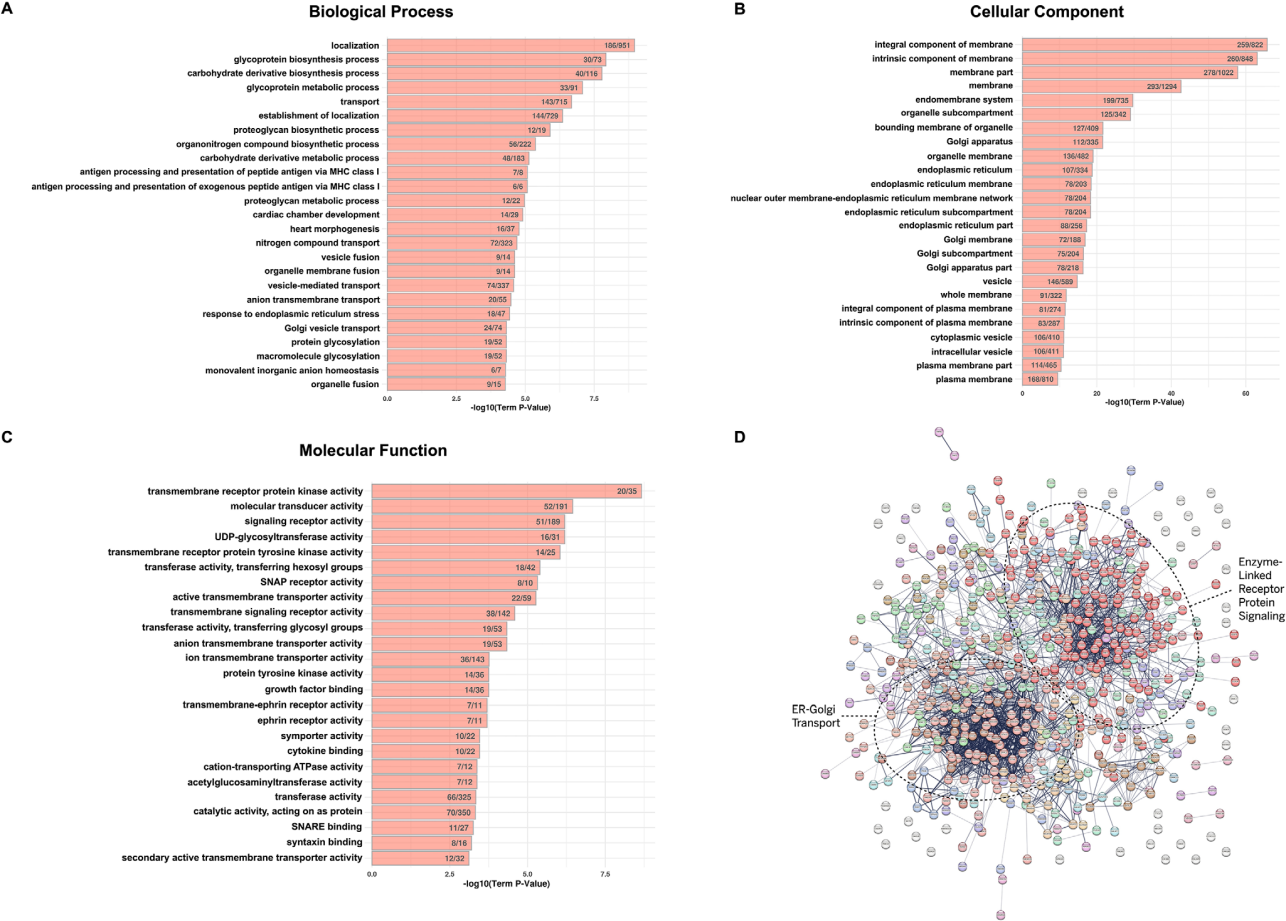
Identification and analysis of the exclusive interactome of GPR161.(A‐C) Gene Ontology analysis of GPR161 exclusive interacting proteins. Top 25 protein categories for Biological Process (A), Cell Component (B), and Molecular Function ontologies (C). The numbers on each bar graph represent the number of proteins identified within each GO term category, relative to the total number of proteins in that category. Due to violation of the IID statistical requirement, FDR adjustment of p‐values was not performed; therefore, the reported p‐values are unadjusted, and a stringent p‐value threshold (*p* <10^−3^) was applied to each GO term. (D) STRING analysis of GPR161 exclusive interacting proteins. Proteins in the same category are color‐coded. Two main nodes, metabolic process, enzyme‐linked receptor protein signaling, and ER‐Golgi transport, are indicated as circles.

### 3.4 Validation of the Co‐Interactome: FKBP8 Is a Co‐Interacting Protein of FUZ and GPR161

We prioritized the co‐interactome of FUZ and GPR161 using the following three criteria for the validation study. First, it is relevant to primary cilia; second, it is related to neural tube development; and finally, it is a Shh signaling molecule, as both FUZ and GPR161 fall into all three criteria. FKBP8 satisfied all three criteria among co‐interacting proteins of FUZ and GPR161, and FKBP8 was also identified as FUZ interacting proteins in the previous study [14]. First, we validated the biochemical interaction between GPR161 and FKBP8 by performing co‐immunoprecipitation experiments with overexpressed GPR161 and FKBP8 proteins in HEK293 cells. FKPB8 and GPR161 proteins were immunoprecipitated using GPR161 pulldown (Figure 5A) and FKBP8 pulldown (Figure 5B), respectively, confirming their biochemical interaction. We then examined the interactions between all three proteins, FKBP8, FUZ, and GPR161. We co‐overexpressed all three genes, FUZ, GPR161, and FKBP8, and then immunoprecipitated with GPR161. The GPR161‐FKBP8 biochemical interaction was not affected by FUZ overexpression, while all three proteins were in the protein complex (Figure 5C). In addition, we further confirmed the individual biochemical interactions between GPR161 and FUZ (Figure 5D: lanes 1 and 2), and between FKBP8 and FUZ (Figure 5D: lanes 3 and 4). Similarly, the biochemical interaction between FKBP8 and FUZ was not changed by GPR161 co‐expression, while FKBP8 still interacts with GPR161 (Figure 5D: compare between lane 4 and 5). In addition, we predicted the structural features of these interactions using Alpha fold 3 [34]. Consistent with our protein interaction data in Figure 5D, structural interaction interfaces among full‐length of FKBP8, FUZ and GPR161 were not overlapping (Figure S6). However, these predictions were considered to be a low confidence interaction (ipTM: 0.18 and pTM: 0.46 for FUZ and GPR161, ipTM: 0.24 and pTM: 0.41). Taken together, these results validate our proteomics finding that FKBP8 interacts with both FUZ and GPR161. They further suggest that the biochemical interactions among these three proteins are not mutually exclusive, implying that each protein likely engages distinct, nonoverlapping binding domains.

**FIGURE 5:**
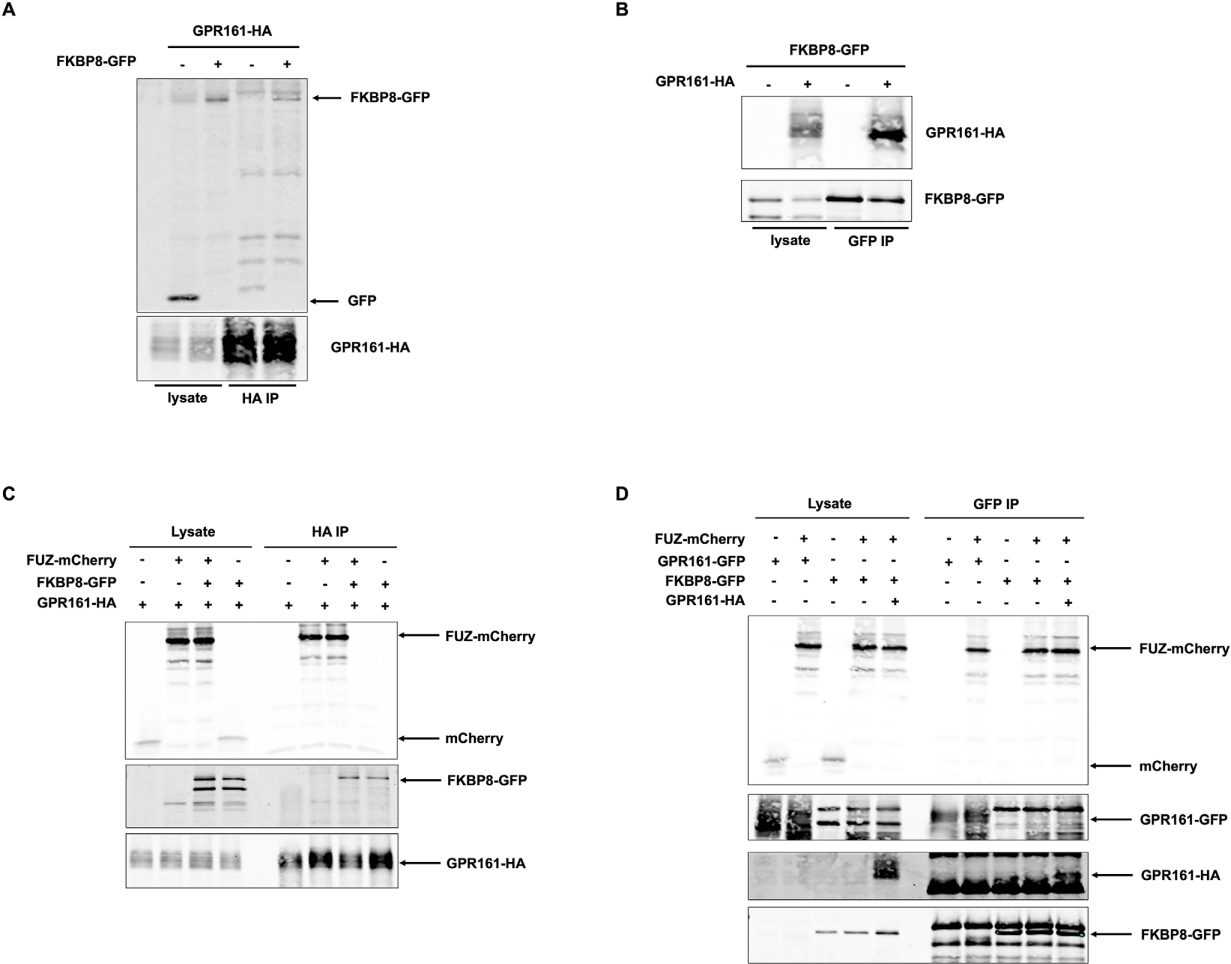
Validation study with a prioritized protein: FKBP8.(A, B) Validation of the biochemical interaction between GPR161 and FKBP8. 293 cells were transfected with FKBP8‐GFP and GPR161‐HA plasmids, and cell lysates were subjected to immunoprecipitation (IP) with an anti‐HA antibody, followed by western blotting (WB) to detect GPR161‐HA and FKBP8‐GFP (A). IP was performed using an anti‐GFP nano‐Trap, followed by WB to detect GPR161‐HA and FKBP8‐GFP (B). (C and D) Biochemical interactions between FUZ, GPR161, and FKBP8. 293 cells were transfected with GPR161‐HA, FKBP8‐GFP, and FUZ‐mCherry plasmids, and the cell lysates were subjected to IP with anti‐HA antibody (C) or anti‐GFP antibody (D), followed by WB to detect GPR161‐HA GPR161‐GFP, FKBP8‐GFP, and FUZ‐mCherry. (A–D) Representative data from *n* = 2 for each IP experiment. IP: Immunoprecipitation.

## 4 Discussion

In this study, we identified the protein interactomes of FUZ and GPR161 to understand their functional relationship using affinity‐based mass spectrometry. Notably, this is the first report characterizing the GPR161 interactome, highlighting the novelty of our work. We then classified the identified interacting proteins using GO and STRING analyses, which revealed both shared and distinct functional roles for FUZ and GPR161. Finally, we prioritized candidate proteins that interact with both FUZ and GPR161, experimentally validated these interactions, and characterized their interaction properties.

Two notable protein categories in the co‐interactome of FUZ and GPR161 are protein catabolism and trafficking. Within protein catabolism, we observed both the proteasome complex and ERAD proteins and E3‐ligases as two large nodes from the co‐interactome (Figure 2), suggesting that FUZ and GPR161 can be regulated by ubiquitin‐dependent degradation for proteostasis regulation. We ruled out the possibility that it could be an overexpression artifact and nonspecific binding with abundant proteins for two reasons: (1) we removed the interacting proteins of the GFP control from the FUZ and GPR161 interactomes, which used the same overexpression system and affinity purification methods, and (2) milder overexpression with lentivirus infection. Remarkably, GPR161 is known to be ubiquitinated at Lys63 for its removal from cilia mediated by the BBSome, with the coupling of endosomal sorting required for transport (ESCRT)‐0 protein [38]. This work is aligned with our previous finding that FUZ, as one of the retrograde intraflagellar (IFT) machinery, might be involved in b‐arrestin‐mediated ciliary removal of GPR161 [21]. A recent publication further supports that both BBSome and IFT machinery mediate b‐arrestin‐dependent ciliary removal of GPR161 [39]. While the ubiquitination of GPR161 at Lys63 is linked to BBSome‐dependent GPR161 ciliary removal, the involvement of IFT in this process remains unclear. Our study provides evidence that FUZ, an IFT machinery component, may serve to remove ubiquitin‐tagged GPR161 from cilia. In addition, the proteomics data that we identified further provide information regarding specific ubiquitin ligases and the potential link between ciliary removal of ubiquitinated GPR161 and its degradation, both of which could be mediated by FUZ. This will be a compelling follow‐up study.

Among the trafficking categories, ciliary trafficking is a particular area of interest given that FUZ and GPR161 are both known to have critical roles within primary cilia. We identified 18 proteins related to ciliary trafficking, including ARL1, ARL6IP5, BAG6, DNAJB6, EXOC4, FKBP8, IGF2R, KIFC2, KTN1, NUP210, NUP93, PEX1, PRKAR1A, PRKAR1B, PSMD2, RAB14, UGGT1, and VCP, of 32 proteins categorized to trafficking proteins (Supporting Information Table). Among them, FKBP8 was of particular interest since FKBP8 has a role in Shh signaling and an implication in cilia (both motile and nonmotile) [40, 41]. Indeed, FKBP8 has been identified as an interacting partner of Ankmy2 [42], which binds to multiple adenylyl cyclase (ACs) that function downstream of GPR161 [43]. In addition, FKBP8 regulates apoptosis and autophagy through mitochondria quality control [44, 45]. This finding further supports a potential connection between FKBP8 and FUZ, as mitochondria related proteins were enriched in FUZ‐ elusive interactome, identified by STRING analysis (Figure 3). Furthermore, FKBP8 functions as a chaperone protein that helps maintain proteostasis [46, 47], suggesting that it may regulate the stability and folding of GPR161 and FUZ. Consistent with this idea, GO analysis of the GPR161‐FUZ co‐interactome revealed enrichment of proteostasis‐related processes (Figure 2B). Lastly, given the established genetic role of *GPR161*, *FUZ*, and *FKBP8* in the pathogenesis of spina bifida in both humans and mice [1, 48], it is plausible that these genes also interact genetically during spinal neural tube development. This possibility warrants further investigation in the future study.

The exclusive FUZ interactome suggests the potential novel functions of FUZ during mitosis. Taken into account its localization in the basal body of primary cilia, the basal body is a remnant of centriole, which plays a critical role in organizing the mitotic spindle of the chromosome during mitosis. Our data (Figure 3) supports the notion that FUZ interacts with proteins related to mitosis, which might lead to its functional role during cell cycle progression. In addition, ubiquitin ligase complex/binding was identified as a FUZ interactome by GO analysis (Figure 3A–C), suggesting that FUZ may be regulated by ubiquitin‐dependent degradation machinery. This novel protein regulatory mechanism of FUZ has implications for birth defects considering that the loss‐of‐function mutations of FUZ are associated with NTDs and ciliopathies [17, 49, 50].

The exclusive GPR161 interactomes included two major categories of proteins: enzyme‐linked receptor protein signaling and ER‐Golgi transport (Figure 4). Considering that GPR161 is a GPCR, the largest receptor family, it is reasonable to identify multiple other receptors for signaling. These results suggest that GPR161 may form protein complexes with other receptors in other signaling pathways, thereby potentially regulating other signaling pathways. Indeed, our previous studies [6, 7, 8] suggest that GPR161 regulates not only Shh but also Wnt signaling, although the molecular regulatory mechanisms have not been fully elucidated. These data provide potential mechanistic insights into our observations. The other remarkable nodes of the STRING analysis with the GPR161 exclusive interactome were ER‐Golgi transport. While GPCR is well known to transport from ER to Golgi, then to plasma membrane, our result showed the specific protein complex for ER to Golgi transport for GPR161. In addition, Popa et al. [51] revealed that GPR161 is a Golgi‐resident protein that maintains the Golgi structure. Our results are also aligned with this notion, suggesting that GPR161 is not only transported via ER‐Golgi transporters but also regulates Golgi homeostasis for its proper secretion and that of other proteins.

Overall, our proteomics study identified the protein network of FUZ and GPR161, which sheds light on the novel functions of FUZ and GPR161, either together or individually. Although overexpression systems are widely used in affinity‐based proteomic approaches [52, 53], we acknowledge the limitations associated with the overexpression system used in this study. Despite this limitation, our study provides molecular insights that advance the understanding of the functions of FUZ and GPR161 and may facilitate the identification of novel diagnostic markers relevant to diseases associated with these proteins.

## Author Contributions

**S. E. K**.: Conceived the overall study. **G. S. and S. E. K**. performed the overall experiments; and **A. B., G. S., and A. J. K**. analyzed the proteomics dataset; **G. S. and S. E. K**. drafted the manuscript, and all authors reviewed and edited the manuscript.

## Funding

This work was supported by grants from the NIH (HD093758 and HD116028) to Dr. Kim.

## Conflicts of Interest

The authors declare no conflicts of interest.

## Associated Data

Mass spectrometry raw and processed data was submitted to MassIVE (MSV000098847) and will be publicly available upon publication.

## Supporting information




**Supporting File 1**: pmic70164‐sup‐0001‐SuppMat.docx.

**Supporting File 2**: pmic70164‐sup‐0002‐SupportingTable.xlsx.

## Data Availability

The data that support the findings of this study are openly available in MassIVE at https://massive.ucsd.edu/ProteoSAFe/static/massive.jsp, reference number MSV000098847.
